# A rumor spreading model based on information entropy

**DOI:** 10.1038/s41598-017-09171-8

**Published:** 2017-08-29

**Authors:** Chao Wang, Zong Xuan Tan, Ye Ye, Lu Wang, Kang Hao Cheong, Neng-gang Xie

**Affiliations:** 1 Department of Mechanical Engineering, Anhui University of Technology, Anhui Ma’anshan, 243002 China; 2 Yale University, New Haven, CT 06520 United States; 3 Engineering Cluster, Singapore Institute of Technology, 10 Dover Drive, Singapore, 138683 Singapore

## Abstract

Rumor spreading can have a significant impact on people’s lives, distorting scientific facts and influencing political opinions. With technologies that have democratized the production and reproduction of information, the rate at which misinformation can spread has increased significantly, leading many to describe contemporary times as a ‘post-truth era’. Research into rumor spreading has primarily been based on either model of social and biological contagion, or upon models of opinion dynamics. Here we present a comprehensive model that is based on information entropy, which allows for the incorporation of considerations like the role of memory, conformity effects, differences in the subjective propensity to produce distortions, and variations in the degree of trust that people place in each other. Variations in the degree of trust are controlled by a confidence factor *β*, while the propensity to produce distortions is controlled by a conservation factor *K*. Simulations were performed using a Barabási–Albert (BA) scale-free network seeded with a single piece of information. The influence of *β* and *K* upon the temporal evolution of the system was subsequently analyzed regarding average information entropy, opinion fragmentation, and the range of rumor spread. These results can aid in decision-making to limit the spread of rumors.

## Introduction

Social communication plays a significant role in the ecology of human beings, and the spreading of rumors is no exception. Widespread rumors can shape public opinion in a country^1^, affect election results or influence the direction of financial markets^2, 3^. The content of rumors can vary from unsophisticated gossip to aggressive marketing material. The social networks of customers may also be exploited by companies to promote their products or services via ‘word-of-email’ and ‘word-of-web’^4^. Additionally, rumor spreading mechanisms can have technological applications. For example, rumor-mongering can form the basis for a class of communication protocols, known as the gossip algorithms. These algorithms have applications in information dissemination and in peer-to-peer file sharing^5, 6^. The conversational properties of rumors have also been analyzed to provide a social explanation for their pervasiveness^7^. A realistic model for the spread of rumors will thus have major theoretical and practical significance, for example, in minimizing damages caused by rumors, regulating the spread of misinformation during a time of crisis, and disseminating relevant news.

Daley *et al*. introduced a standard model of rumor spreading, known as the Daley–Kendal (DK) model^8, 9^. The DK model and its variants, such as the Maki–Thompson (MK) model^10^, have been used extensively in the study of rumor spreading. Readers can refer to refs 8–10 for more information. With the development of complex networks^11, 12^, rumor propagation dynamics also included the topology of social networks. Moreno *et al*. investigated the stochastic version of the MK model on scale-free networks by using Monte Carlo simulations^13^. Nekovee *et al*. improved the MK model by formulating the model on networks in terms of interacting Markov chains (IMC) and derived mean-field equations that describe the dynamics of the model on complex social networks^14^. Boccaletti *et al*. investigated the influence of the source on the propagation dynamics by studying the effect of hub nodes on propagation ability^15^. They concluded that the propagation ability of hub nodes is strong, but has weak stability. Drawing from epidemic theory, Dotts *et al*.^16^ then introduced a general model of contagion which, by explicitly incorporating memory of past exposures into the susceptible-infected-removed (SIR) model, included the main features of existing contagion models and could interpolate between them. From such models, Zhao *et al*.^17, 18^ proposed a susceptible-infected-hibernator-removed (SIHR) model which incorporated the mechanisms of memory and forgetting, while Lü *et al*.^19^ studied the strengthening of multiple infections and the influence of the external social factors in the established transmission model.

Research into the evolution of public opinion is of mutual relevance to models of rumor spreading and information exchange since pieces of information shared by multiple people can also be thought of like opinions. In the Sznajd model^20^, magnetization and phase change phenomena of ferromagnetic materials were used to simulate the evolution of binary opinions. The model has since been extended to two-dimensional lattices, small-world networks, and scale-free networks^21–23^. Hegselmann R. *et al*.^24^ presented a model based upon bounded confidence, in which individuals would only take into account other opinions that were within a certain confidence of their opinions, and ignore opinions outside that range. Chen *et al*.^25, 26^ proposed a majority decision model, where the probability of a subject’s belief in an opinion was proportional to the number of agents in the neighborhood who held that opinion. Zanette *et al*.^27^ approached the topic by considering the heterogeneity of the population properties and proposed a model for the co-evolution of opinions and network topology. Based on this framework, a new model for the synchronized evolution of public opinions and networks was proposed by Fu *et al*.^28^ which incorporated the principles of majority preference and minority-avoidance. Other approaches include research by Martins *et al*.^29, 30^, who applied the Bayesian process to model the exchange of individuals’ opinions and Mare *et al*.^31^, who studied an opinion persuasion mechanism based on game theory.

It is also known that distortion during the process of spreading and the spread of distortions thus produced are vital aspects in the transmission of rumors. When a rumor spreads, states of panic, coupled with failures in the mechanisms of transmission or cognition, may cause the communicated information to be distorted. The ‘trembling hand’ effect^32^, whereby individuals cannot control exactly how they reproduce rumors, can also play a part^33^. Thus, mistakes inevitably appear. Whether intentionally or unintentionally, existing information becomes distorted, and new information is formed and spread.

In this paper, we present the first comprehensive model that captures the role of memory, conformity effects, differences in the subjective propensity to produce distortions, and variations in the degree of trust that people place in each other, through the concept of information entropy—a measure of the chaos or noise of a particular set of data. Unlike the previous models described above, our model is now able to provide a more realistic description of the process by which rumors are propagated and public opinion formed. People have a certain capacity for remembering pieces of information, and the noice of that memory can be expected to influence the rate at which they distort information or make mistakes. Conformity effects can also be accounted for as a process whereby individuals spread the pieces of information that are most salient within their memory, that is they uncritically repeat what they have previously heard. On top of that, network topology plays a role in rumor acceptance, since people do not wholeheartedly believe all new information they receive, but trust others based on their relative positions within the social network^33^.

## The model

Individuals are modelled as nodes in a BA scale-free network of size *N*, and the spread of information is modelled as occurring in three consecutive phases: spreading, acceptance, and updating. In the following sections, we first define the relevant variables and parameters, before describing each phase in detail.

### Information representation

For simplicity of analysis, each piece of information is defined as a binary string of length *s* (e.g. ‘11011’ when *s* = 5). Since information is binary, there are 2^*s*^ possible different types of information, where each type is labelled with an integer 0 ≤ *i* ≤ 31. Because the model is so general, each information type can alternately be viewed as a piece of news, an opinion, or a (potentially distorted) fact.

### Memory capacity

Individuals can remember information, and we assume that every individual has the same memory capacity, *L*. That is, each individual can remember up to *L* pieces of information.

### Information salience

Within the memory of person *n*, the frequency of occurrence of the *i* ^th^ type of information is denoted by *f*_*i*_. This provides a measure of the salience of that type of information – if *f*_*i*_ is high, it means the person has received that type of information many times from others, marking it as highly salient. By having individuals repeat the information that is most salient in their memory, as is described later in the spreading phase, conformity effects can be accounted for.

### Information entropy

*Hn* denotes the classical Shannon information entropy for individual *n*, defined as:

1 $${H}_{n}=-{\sum }_{i}{f}_{i}{\mathrm{log}}_{2}{f}_{i}$$

where *f*_*i*_ is the frequency of information type *i*, defined earlier. Entropy increases as the distribution of different types of information becomes more uniform (*f*_*i*_ → 1/2^*s*^), but decreases as one type of information begins to dominate (*f*_*i*_ → 1), reflecting the greater amount of chaos and noise that an individual has to contend with as they remember a wider variety of information.

### Probability of distortion

Our model assumes that the propensity for each individual to distort information is related to the entropy of the information stored in their memory. The greater the entropy, the more uncertain their memory is, and the more likely they are to make errors when recalling and reproducing information. The probability of information distortion by individual *n* is thus defined as

2 $${P}_{n}=\frac{1}{\exp (\frac{{H}_{{\rm{\max }}}-{H}_{n}}{{H}_{\max }}K)+1}$$

where *K* ≥ 0, the *conservation factor*, represents the control force against information distortion, and *H*_max_ is the maximum possible information entropy (i.e. when *f*_*i*_ = 1/2^*s*^). When *K* is large, the ability to control information distortion is strong, and vice versa. As *H*_*n*_ approaches *H*_max_, the probability of distortion approaches a maximal value of ½, whereas as *H*_*n*_ approaches zero, the probability of distortion approaches a minimal value of 1/(*e* ^*K*^ + 1).

### Probability of acceptance

When a person *m* receives a piece of information from another person *n*, person *m* will not always believe the information received. Rather, acceptance of information depends on how trustworthy person *m* considers person *n*, which in our model is based upon the relative social status (as measured by number of connections) of person *n* among the neighbors of the receiver. The more trustworthy *n* is, the higher the probability *η*_*mn*_ that person *m* will accept the information, where the probability of acceptance *η*_*mn*_ is given by

3 $${\eta }_{mn}=\frac{{k}_{n}^{\beta }}{\mathop{\max }\limits_{l\in nbd(m)}({k}_{l}^{\beta })}$$

in which nbd(*m*) denotes the set of *m*’s neighbors, *k*_*n*_ denotes the degree of node *n* (i.e. the number of connections that person *n* has with other people), *k*_*l*_ denotes the degree of each neighbor *l*, and *β* is a parameter called the *confidence factor*. If *β* > 0, this means that person *m* will trust neighbors with more connections, and vice versa. In the special case where *β* = 0, then *m* will trust all neighbors equally (i.e. accept information from each of them with equal probability).

### Spreading phase

An individual *n* begins to disseminate information in the spreading phase. Out of all the strings of information currently remembered, the most salient type of information *i* (i.e. the type *i* that occurs with the highest frequency *f*_*i*_ within *n*’s memory) is selected for transmission, with random arbitration should there be two or more types of information with maximum saliency. With probability *P*_*n*_, this string of information becomes distorted through bitwise mutation at a randomly selected point (‘1’ is flipped to become ‘0’, and vice versa). This mutation occurs within *n*’s memory – specifically, the *first* piece of information of type *i* in *n*’s memory is mutated to become the new type *j*. The potentially distorted piece of information is then spread to all of *n*’s neighbors. If individual *n* currently has no information in memory, then they will not participate in information spreading.

### Acceptance phase

When each neighbor *m* receives this new piece of information from *n*, it will selectively accept or reject it. Acceptance occurs with a probability of *η*_*mn*_, which, as defined earlier, depends on how much trust agent *m* gives to agent *n*. If *m* accepts, the new piece of information is added to *m*’s memory bank.

### Updating phase

Since each person has a finite memory capacity, the memory bank acts like a first-in-first-out (FIFO) queue, with newly accepted pieces of information displacing the oldest pieces within memory once capacity has been reached. Furthermore, the updating of memory banks occurs synchronously across individuals: at every time-step *t* of the process, *all* individuals try to spread the most salient type of information currently known by them at time *t* to their neighbors, and then all individuals synchronously decide whether to accept or reject new information received from their neighbors, after which the memory banks are updated to reflect a new set of values at time *t* + 1. For example, if person *n* has five neighbors, person *n* will try and spread one piece of information to each them, potentially distorting it, and receive five pieces of information in return. Some of these pieces will be rejected; perhaps only two are accepted. These two pieces of information will enter person *n*’s memory bank, and if *n* was already at maximum memory capacity, the two oldest pieces in the bank will be forgotten.

## Results

Computer simulations were performed to investigate the model presented here. To ensure sufficient diversity of information types, the length of the information bit-strings was set to *s* = 5, giving 2^5^ = 32 types in total. The Barabási–Albert (BA) scale-free network^12^ was initialized with *m*_0_ connected nodes. New nodes were added to the network one at a time. Each new node was connected to *m* existing nodes with a probability proportional to the number of links that the existing nodes already have. The total number of nodes was *N* = 3000, with *m*_0_ = 5, *m* = 2 as the other network parameters. Following the generation of the network, a random individual was selected to be the initial propagator, with only one string of information in its memory: ‘00000’. All other individuals started with empty memory banks. Since rumor propagation is affected by the memory capacity *L*, a suitable value needed to be determined. After several calibration trials, a capacity of *L* = 320 was selected (law of large numbers applies). Our simulations also predict that similar results can be obtained for *L* > 320.

In the subsequent sections, the time-evolution of the network is analyzed in terms of several different metrics: average information entropy, opinion fragmentation, and the range of information spread. This is followed by an analysis of the steady-state characteristics of rumor propagation.

### Average information entropy

The average information entropy of the population $\bar{H}$, reflects the level of information noise in the entire system. A higher average entropy corresponds to a more uncertain network where rumors and distorted information are abundant. $\bar{H}$ is defined as follows:

4 $$\overline{H}=\frac{1}{N}\sum _{n}{H}_{n}$$

**Figure 1:**
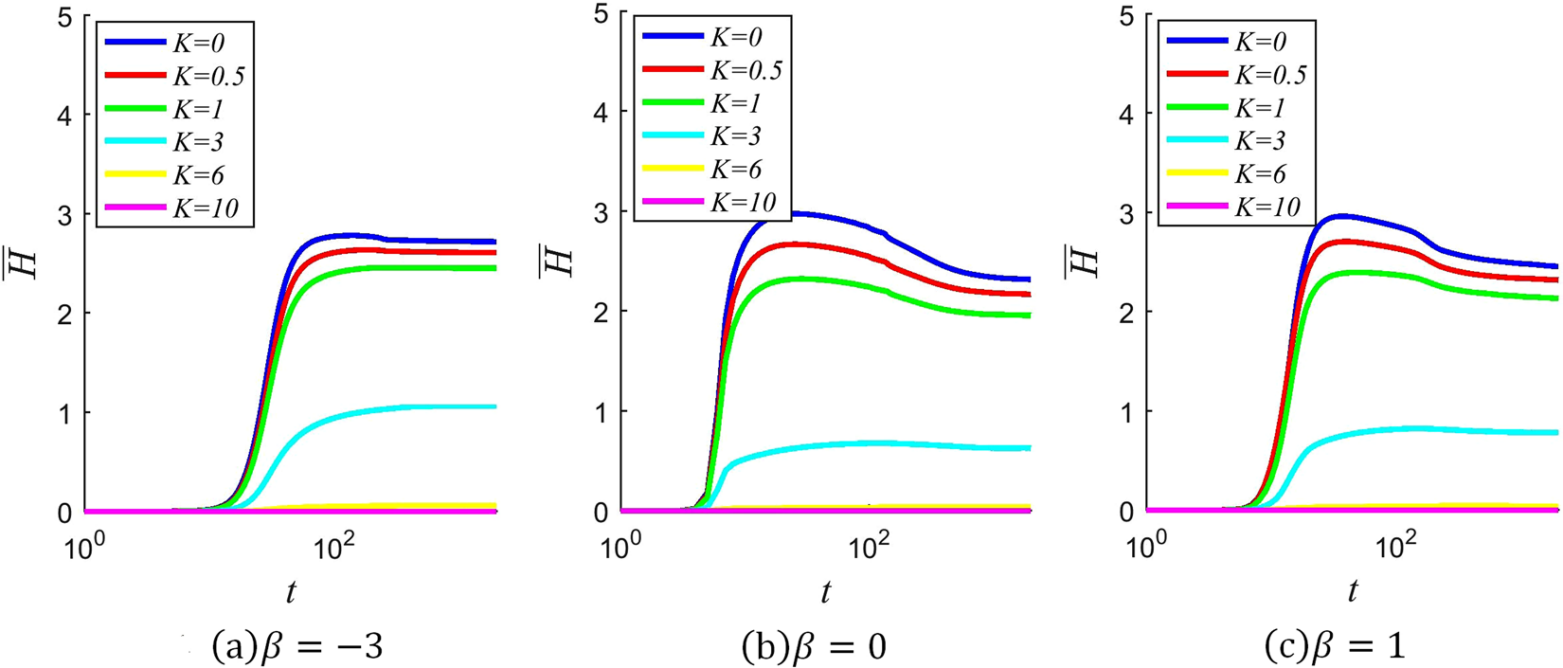
Evolution of average entropy over time for different values of *β* and *K*. (The curves are obtained by averaging over 10^3^ simulations with the same initial conditions and parameters, on the same generated network).

The evolution of the average entropy of the network over time is shown for several different values of *β* and *K* in Fig. 1, where the x-axis denotes time (i.e., the number of iterations in the simulation). It can be observed from the Figure that in most cases, an entropy explosion occurs sometime between *t* = 10 and *t* = 100, with the average information entropy shooting upward rapidly. The effect of varying *β*, the confidence factor, is significant but not very pronounced. In the case where *β* = −3, the average entropy stabilizes at a higher level than in the other two cases. When *β* = 0 or *β* = 1, the entropy shoots up to approximately the same value, before gradually decreasing and stabilizing at similar levels.

On the other hand, the effect of varying *K*, the conservation factor, can be seen to have drastic effects. When *K* is sufficiently large, as in the case where *K* = 10, the entropy explosion does not happen at all, and the average information entropy stabilizes at a level very close to zero. In the cases where *K* ≤ 1, however, the sharp increase in entropy occurs with approximately the same temporal dynamics, with the final average entropy only increasing slightly with a decreasing value of *K*.

This nonlinear response to the value of *K* can be explained as follows: Since the initial propagator only possesses one type of information (‘00000’), which corresponds to an entropy of *H*_*n*_ = 0 and a distortion probability of *P*_*n*_ = 1/(*e* ^*K*^ + 1), a large value of *K* results in a *P*_*n*_ that is very low. As a result, the information that is spread during the initial stages has a very low chance of distortion, which in turn ensures that the information entropy of recipients is kept low, since they mostly end up possessing only the initial undistorted piece of information. These effects compound, such that over a long period time, the uncorrupted information still prevails, and rumors are close to absent. But when *K* is low, the effects compound in the opposite direction – a higher initial probability of distortion results in higher entropy during the initial stages, and so on – such that eventually a sharp increase in average entropy occurs.

The results thus demonstrate the importance of the conservative factor *K*. In a vigilant community where individuals are very careful to guard against distortion, rumors do not spread, but if the community is just slightly more prone to mistakes and fabrications, then rumors and misinformation are bound to break out on a wide scale.

### Opinion fragmentation

In this section, we use the term ‘opinion’ as a more natural way of describing information or beliefs held by individuals that may end up differing from the beliefs of others over time. With respect to our model, we define an individual to hold the opinion *i* if the most salient piece of information in their memory is of type *i* (i.e. the piece of information with maximal *f*_*i*_). Let *D*_*i*_ be the total number of individuals with opinion *i*. Then we can also define

5 $${\delta }_{i}={D}_{i}/N$$

as the proportion of individuals who hold the opinion *i*. By analyzing how *δ*_*i*_ changes for each opinion over time, we can study the fragmentation of opinion across the entire population.

**Figure 2:**
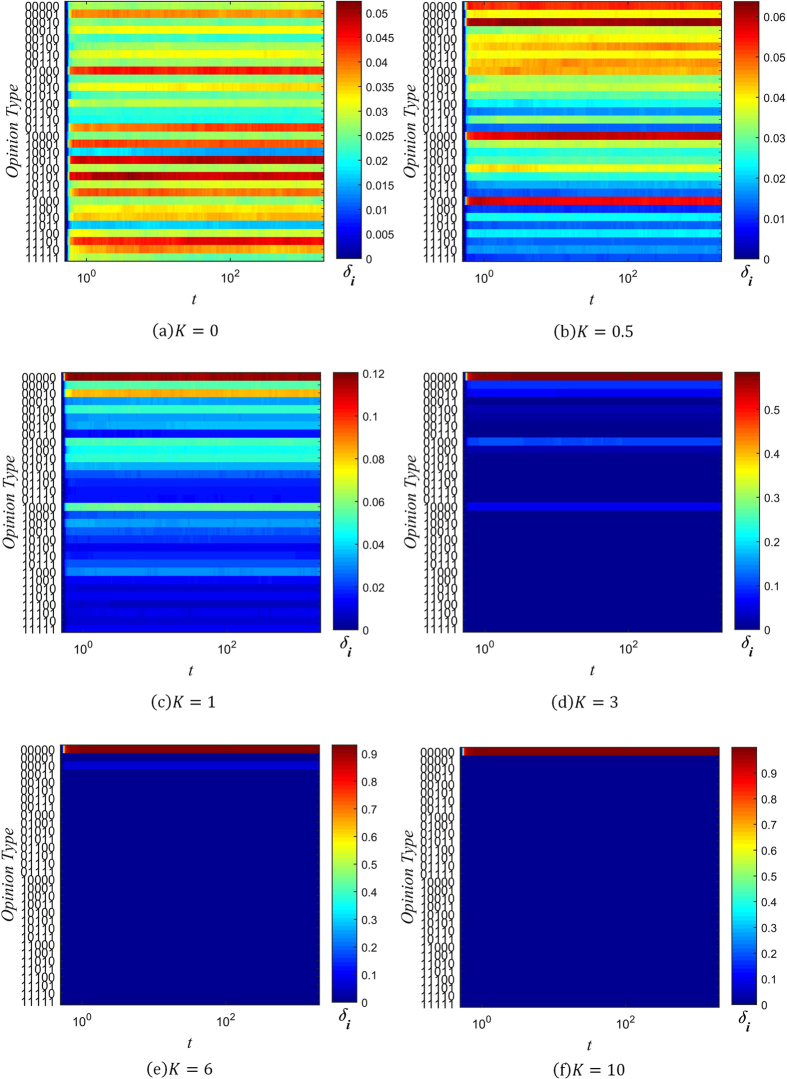
Evolution chart of *δ*_*i*_ (*β* = −3).

**Figure 3:**
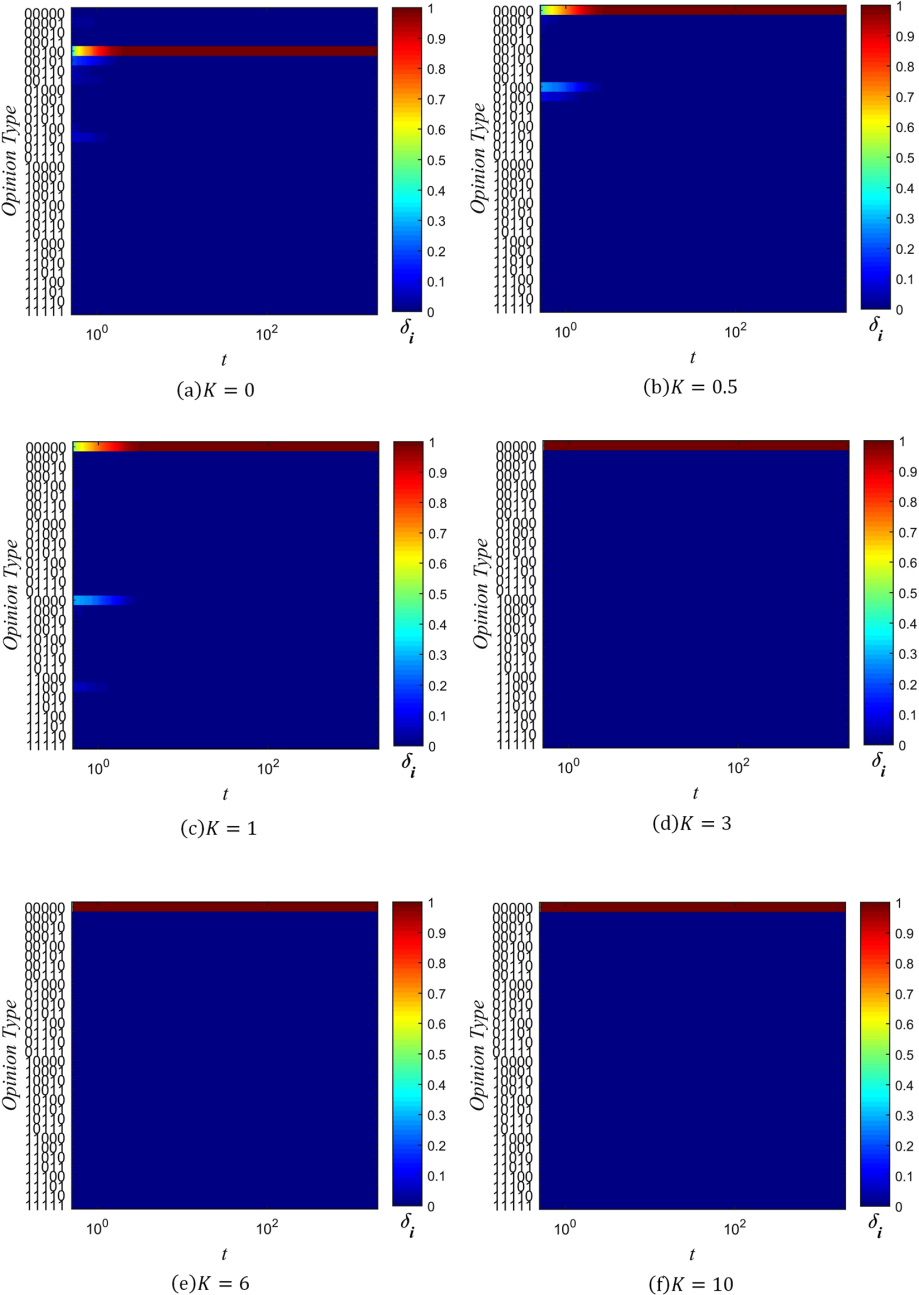
Evolution chart of *δ*_*i*_ (*β* = 0).

**Figure 4:**
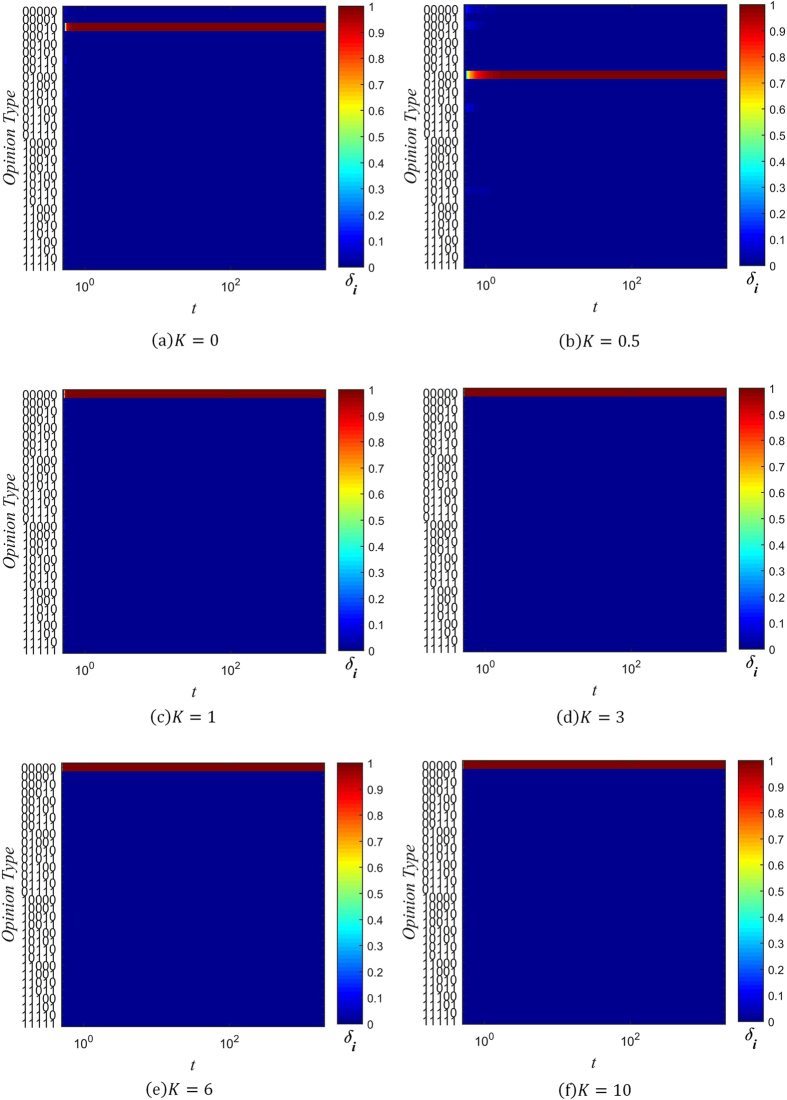
Evolution chart of *δ*_*i*_ (*β* = 1).

Figures 2 to 4 below show the fragmentation of opinion over time for several values of *β* and *K*. The x-axis of each graph denotes time, and the y-axis denotes the string of information corresponding to each opinion type *i*. The value of *δ*_*i*_ is represented by color, with red corresponding to a higher proportion of individuals holding that opinion, and blue corresponding to a lower one.

When *β* = −3, individuals trust other individuals who are *less* well connected. As can be seen from Fig. 2, this results in large degree of opinion fragmentation. In Fig. 2(a) to (c), where *K* ≤ 1, every type of opinion rapidly ends up with some level of popular support, despite there being only one initial seed opinion (*i* = 0, corresponding to the string ‘00000’). As one might expect, the fragmentation is less severe with increasing values of the conservative factor *K*. When *K* = 0, support for different opinions is more or less equally spread out, but as *K* increases to 1, opinions are clustered around the initial opinion of ‘00000’, which remains the most popular opinion. As *K* increases further, the clustering around the initial opinion becomes more pronounced, with little opinion fragmentation when *K* = 3, and even less when *K* = 6. Finally, when *K* = 10, this is sufficiently high that initial opinion type dominates the network, and there is virtually no fragmentation.

The case where *β* = 0 corresponds to the situation where individuals trust all their neighbors equally. It can be seen from Fig. 3 that a dominant opinion eventually forms in this situation for all six values of *K* simulated. We call this dominant opinion the public opinion. When *K* = 0, the public opinion is ‘00100’, which differs from the initial opinion type 0. However, when *K* ≥ 0.5, the public opinion is always the same as the initial seed opinion.

Lastly, Fig. 4 shows that when *β* = 1, a dominant opinion forms after the evolution of the system for all six values of *K*. This corresponds to the case where individuals trust others who are *more* well-connected. When *K* = 0 and *K* = 0.5, the public opinion (‘00010’ and ‘01000’ respectively) differs from the initial information type. For the other four cases, the public opinion is the same as the initial opinion.

A general trend that can be observed is that opinion fragmentation decreases as the values of *β* and *K* increase. The effect of increasing *K* is due to *K*’s role in limiting information distortion, as explained in the previous section. With less information distortion, fewer opinion types get generated, resulting in less opinion fragmentation. The effect of increasing *β* can be understood intuitively as follows: When *β* is high, well-connected individuals are more trusted, and so dominant opinions propagate more rapidly, since these individuals will share their opinion with their many neighbors, who are highly likely to accept their opinion. When *β* = 0, dominant opinions still have natural advantage, because well-connected individuals are more like to hold these opinions, and consequently spread them.

On the other hand, when *β* is less than zero, there is a bias against accepting opinions held by well-connected individuals, and towards opinions held by more isolated individuals. Since more isolated individuals are in less contact with any opinion groups that may be initially dominant, they are more likely to hold less common opinions, which they then spread to others. Because of this selection pressure towards less common opinions, negative values of *β* easily result in opinion fragmentation.

### Range of information spread

**Figure 5:**
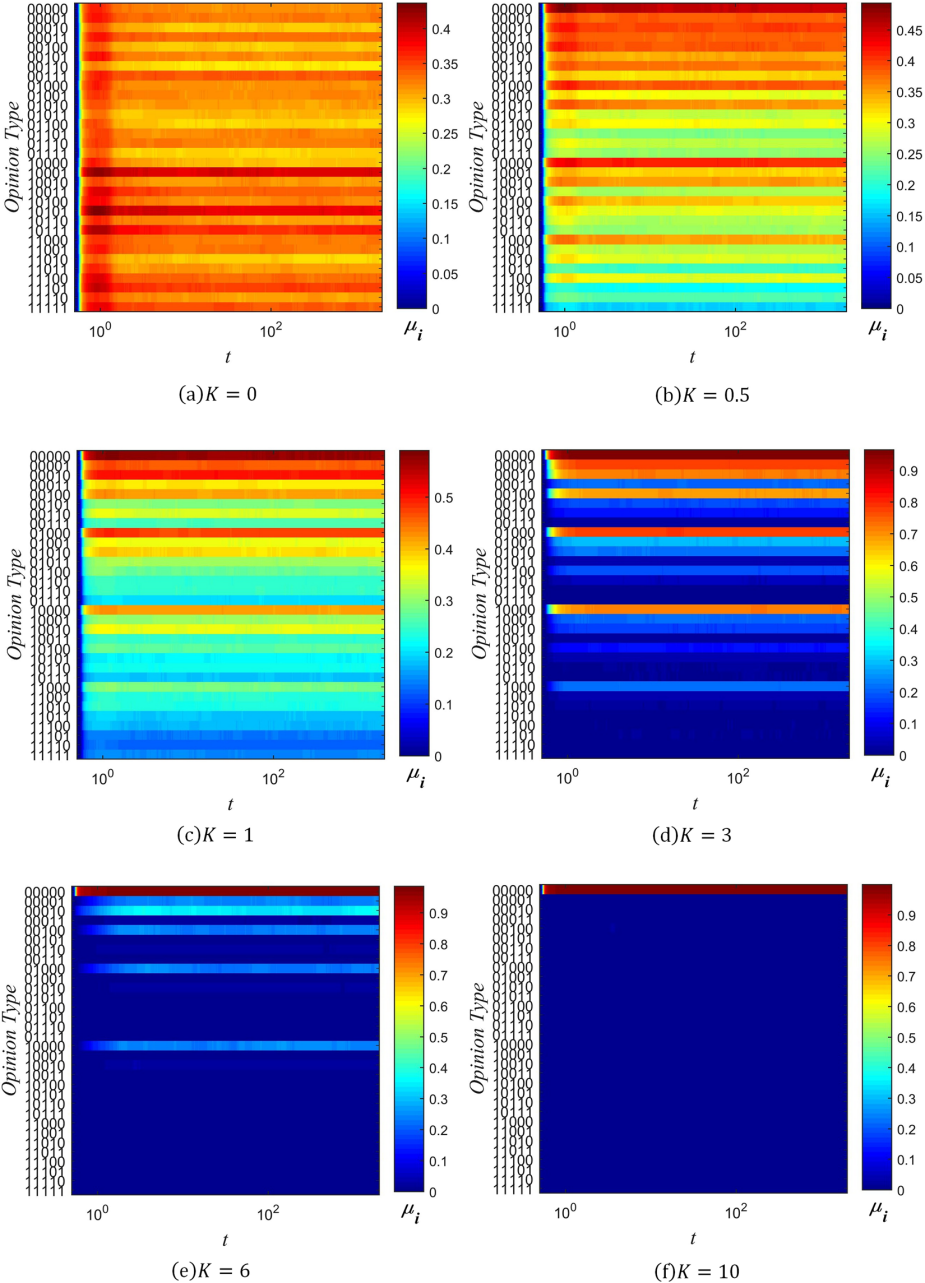
Evolution chart of *μ*_*i*_ (*β* = −3).

**Figure 6:**
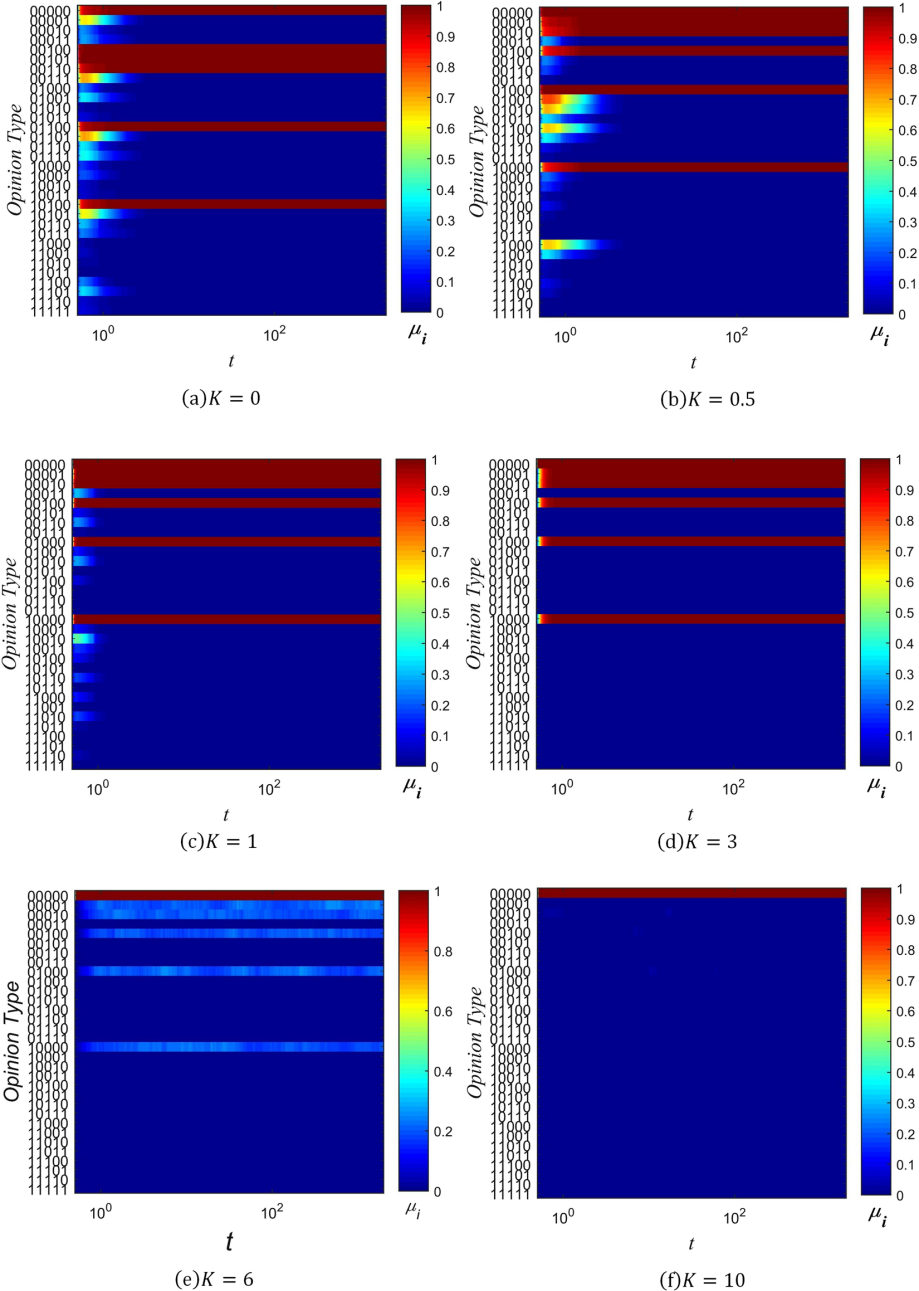
Evolution chart of *μ*_*i*_ (*β* = 0).

**Figure 7:**
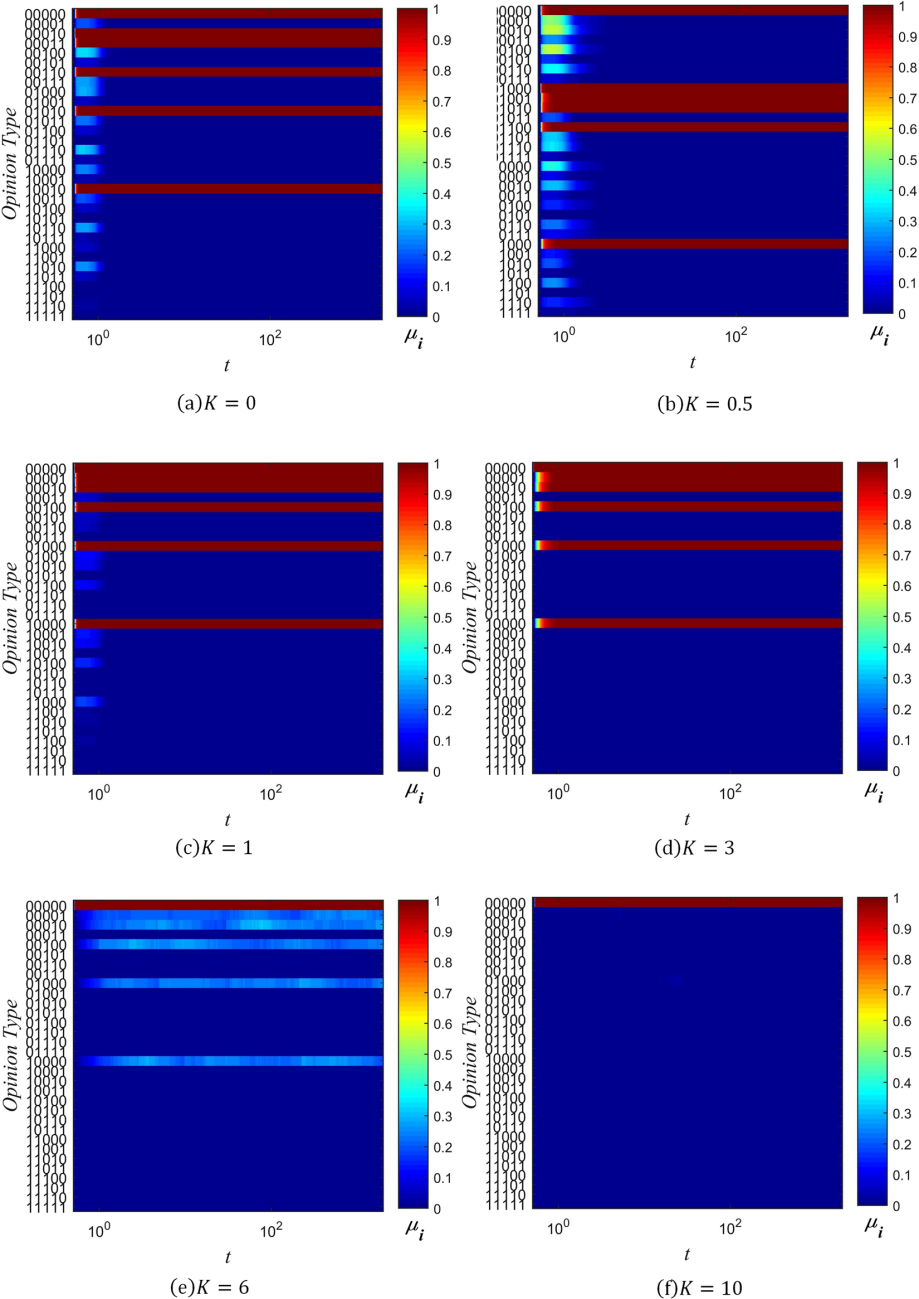
Evolution chart of *μ*_*i*_ (*β* = 1).

Another metric that can be used to analyze the spread of information or rumors is the range of spreading, i.e., the number of individuals in the network who end up hearing a rumor. More precisely, we define *W*_*i*_ as the number of individuals with information type *i* present in their memory banks, and

6 $${\mu }_{i}={W}_{i}/N$$

as the range of information spread, normalized to the total number of individuals in the network. Figures 5, 6 and 7 show the changes in the distribution of *μ*_*i*_ over time for several values of *β* and *K*, with color used to represent the value of *μ*_*i*_.

When *β* = −3 and *K* ≤ 1, the spreading of rumors is rampant, as can be seen in Fig. 5(a)–(c). The diversity of information types in the network quickly saturates, with all 32 possible types gaining a positive value of *μ*_*i*_ in a short amount of time. As the value of *K* increases, the distribution of *μ*_*i*_ coalesces around the initial information type, since the probability of distortion is lowered. At *K* = 3, there end up being 6 types of information which are dominant, in that they have significantly larger *μ*_*i*_ than the other types of information. Of these 6 types, the initial type is the most dominant. This trend continues with *K* = 6, except the 5 other types besides the initial type have much lower *μ*_*i*_. Once *K* is sufficiently high, as in Fig. 5(f), the initial type rapidly attains maximal range (*μ*_i_ = 1), while all other types of information are effectively absent (*μ*_*i*_ = 0).

Setting *β* ≥ 0 changes the distribution of *μ*_*i*_ significantly. As can be seen in Figs 6(a)–(d) and 7(a)–(d), when *β* ≥ 0 and *K* ≤ 3, 6 different types of information come to dominate the network, with each of them approaching the maximal range of *μ*_*i*_ = 1. As the value of *K* increases, the non-dominant information types fade into obscurity more rapidly. When *K* = 6, there are still 6 information types which spread across the network. However, just the initial type is dominant, and the other 5 types spread across about only 20% of the network. Finally, when *K* is increased to 10, only the initial information type prevails, as can be seen in Figs 6(f) and 7(f).

It is evident from these charts that when *K* ≤ 1, many distorted versions of the original string appear early on. This is due to the high probability of information distortion. But as long as *β* ≥ 0, the system gradually reaches a stable spreading state with 6 dominant types of information. This can be understood by analyzing the effect of the confidence factor *β* on *μ*_*i*_, which is similar to its effect on opinion fragmentation.

When *β* < 0, individuals with less connections are trusted more, and since they are more likely to hold uncommon pieces of information (due to their isolation from well-connected hubs that might influence them otherwise), the information they hold quickly spreads throughout the network. The rate of spread is further aided by the large number of low-degree nodes present in a BA scale-free network. On the other hand, when *β* ≥ 0, well-connected individuals have an advantage in spreading the information they hold. Under their guidance, less connected individuals eventually take up the dominant information types, and forget the less common information types in their memory. As a result, when *K* ≤ 1, the network reaches a state with only 6 types of information present.

Combining our analysis of opinion fragmentation with this section, it is clear from Figs 2, 3, 4, 5, 6 and 7 that the conservation factor *K* determines the survival of the initial string of information. The larger the value of *K*, the more likely the initial information type will be retained.

### Steady-state characteristics of rumor propagation

**Figure 8:**
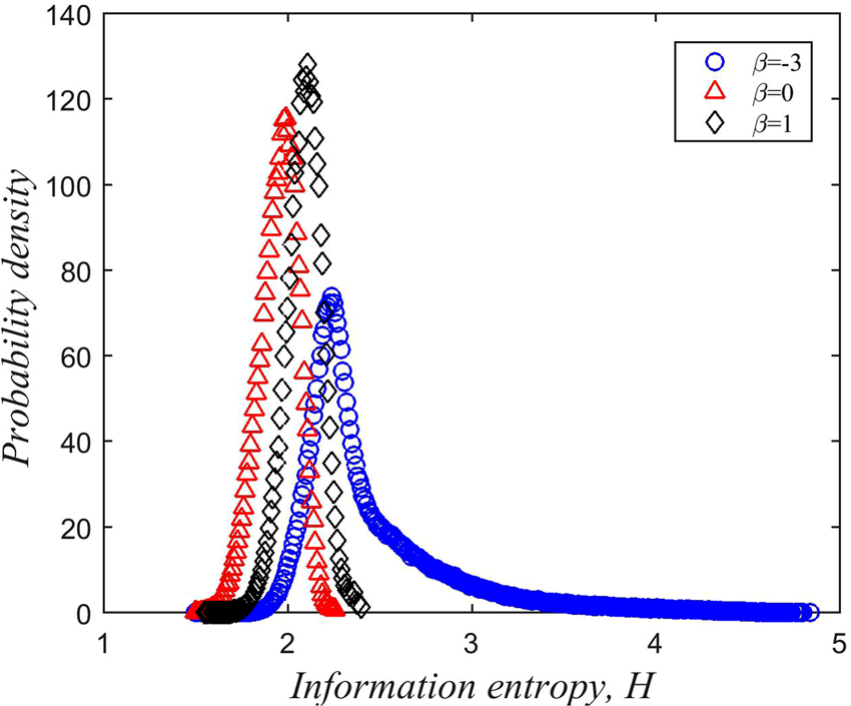
The density distribution of the information entropy of the population (*K* = 1).

**Figure 9:**
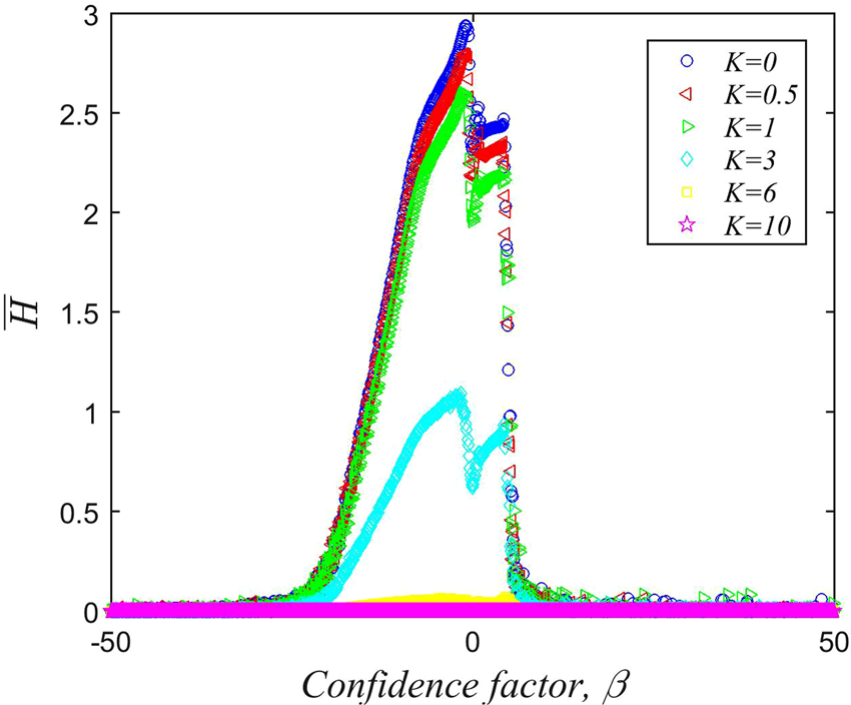
Relationship between *β* and the information entropy of the population.

The steady-state characteristics of our model can be analyzed by using the distribution of information entropy in steady-state (that is, the distribution of information entropy no longer changes). The number of iterations taken was 2000. The curves in Figs 8 and 9 are obtained by averaging over 10^3^ simulations with the same initial conditions and parameters, on the same generated network.

Figure 8 shows the density distribution of the information entropy *H* in the steady-state when *K* = 1. Our simulation results predict that:

1. The distribution of *H* appears to conform to a Poisson distribution;
2. When *β* = −3, the information entropy is much more widely distributed.

The first observation motivates future work as further exploration is beyond the scope of the current study. The second observation (*β* = −3) can be understood using the same reasons that explain opinion fragmentation: When the population places more trust in less connected individuals, all sorts of rumors become prevalent, such that the proportion of individuals with uncertain memory banks becomes larger. As a result, the distribution of information entropy has a fatter tail.

Figure 9 shows the relationship between the confidence factor *β* and the average information entropy $\bar{H}$ in the steady-state for different value of *K*. When *β* < −1, $\bar{H}$ increases as the value of *β* increases. When *β* = −1, $\bar{H}$ reaches a maximum of 2.9. As *β* increases further, a weak collapse of $\bar{H}$ occurs, reaching a local minimum of 2.28 when *β* = 0. After this, a weak upward trend continues until a second collapse occurs at *β* ≈ 5. Following this collapse, $\bar{H}$ stays very close to zero. These trends can be understood as follows:

From Equation (3), we know that as *β* tends to negative infinity, individuals end up trusting only neighbors with the smallest degrees of connection, such that the information they receive becomes highly limited. As a result, most individuals only have one or two types of information in memory, resulting in a low average entropy $\bar{H}$. As *β* increases but remains negative, more small-degree nodes (i.e. individuals with small degrees of connection) become trusted. Since there is a large number of small degree nodes in the network, and since these nodes are more likely to possess uncommon types of information, the information entropy increases rapidly with *β*.

However, when *β* goes from −1 to 0, small-degree nodes begin to lose their advantage over high-degree nodes (i.e. well-connected individuals), who already have a natural advantage in spreading information, and who are more likely to possess common types of information. This results in greater homogeneity of information across the network, and as such there is a weak collapse in the information entropy $\bar{H}$. When *β* begins to increase from 0, there is a certain heterogeneity of information as individuals with a large degree begin to play a larger role, competing with each other to establish their own information type, or opinion, as the dominant one. Thus, $\bar{H}$ grows together with *β* for a while. As *β* continues to increase above 5, individuals begin to trust only neighbors who are extremely well-connected, and the information received again becomes highly limited. Most individuals end up only receiving one type of information from the high degree nodes they are connected to, and so the average information entropy, $\bar{H}$ collapses to 0.

## Discussion

In general, it can be observed that the network’s performance varies similarly with *β* and *K* across all metrics used. Information entropy and rumor spreading tend to increase as the conservative factor *K* decreases. The relationship with the confidence factor *β* is more complex, but it can be seen across metrics that when *β* is negative and small in magnitude, this leads to high levels of information distortion, whereas less distortion is present when *β* = 0 and *β* = 1. We can deduce that rumors are less likely to spread if people are less likely to distort information (higher *K*) or if they are more inclined to trust popular peers with high degrees of connectivity over unpopular ones (*β* ≥ 0).

If we focus only upon the average information entropy, $\bar{H}$ or range of rumor spread, *μ*_*i*_ as metrics, then the results may appear dismal. As Fig. 1 shows, it is difficult to ensure that information entropy remains low unless the conservative factor *K* is at a high value of 10. Similarly, Figs 5, 6 and 7 show that only a high value of *K* ensures that no other information types have a wide spread except the initial one. These simulation results predict that the creation of rumors may be very difficult to stem, since a high value of *K* would require people to have an extremely low probability of distorting the information they receive (for example, if *K* = 10, and an agent only possesses one piece if information, then their probability of distortion *P*_*n*_ = 0.005%).

However, if we look instead at opinion fragmentation as a metric, rumors appear considerably more manageable. As Figs 3 and 4 show, as long as *β* = 0 or *β* = 1, there is only one dominant opinion type in the long run (the opinion type *i* where *δ*_*i*_ = 1). Furthermore, as long as *K* ≥ 1, the final public opinion is equal to the initial ‘correct’ opinion. Even though the average information entropy $\bar{H}$ or range of rumor spread *μ*_*i*_, might be quite high, there might still be little fragmentation of opinions. This is because while each agent may have many different types of information in memory, the most common type of information, which represents the agent’s opinion, can still be the same across all agents. In other words, there may be cases where two persons may each have heard many different versions of the ‘truth’, but the version that they have heard most frequently is common to both of them. Viewed in this light, the existence of other rumors is far less damaging, and the conditions required for public agreement are much less demanding. All we need is that people are reasonably conservative (*K* ≥ 1), and that they tend to trust people with more connections (*β* ≥ 0).

The insights provided by our model have several possible applications. At a rudimentary level, network designers and moderators should try to decrease the probability of distortion (that is, decrease *K*) and increase the amount of trust in high-degree nodes (i.e. increase *β*) if they wish to control the spread of rumors. For example, the probability of distortion could be decreased by education that emphasizes critical thinking, the encouragement of epistemic best practices like independent fact-checking, and the establishment of truth-seeking and rationality as a social norm. However, given that these are long-term solutions that require gradual social change, influencing the level of trust placed in high-degree nodes might yield more immediate results. In the case of Internet platforms, a network architect could establish a mechanism where well-connected and highly active users earn higher trust ratings (in the style of Stack Overflow^34^), or where posts by highly connected individuals appear more frequently in the newsfeeds of followers, thereby giving high-degree nodes a trust advantage and limiting the amount of distortion and fragmentation. Conversely, if architects and moderators wish to prevent groupthink and increase the diversity of ideas, low-degree nodes could be given the advantage of greater publicity.

## Limitations and Future work

The current study is based on BA scale-free network. Future simulations will involve other networks for comparison. It may also be possible to formulate an analytical treatment of the model, similar to approach taken in ref. 35. Whether such analytical functions exist is a subject for future work.

## Conclusion

In this paper, we have considered various factors in the process of rumor spreading, including the role of memory, conformity effects, differences in the subjective propensity to produce distortions, and variations in the degree of trust that people place in each other. The relative strength of these effects was parameterized using the confidence factor *β* and the conservation factor *K*, thereby establishing a realistic and general model of rumor spreading based on information entropy. Our simulation results predict that:

1. For a given confidence factor *β*, the average information entropy decreases with an increase in the conservation factor *K* of the system.
2. When the conservation factor *K* is small and the confidence factor *β* is less than 0, opinions are highly fragmented, and many types of rumor are widespread. On the other hand, when *β* is greater than or equal to 0, the entire population converges upon a single type of information: the public opinion. For a given value of *β*, the value of *K* determines the survival of the initial information type, with larger *K* resulting in a greater probability of survival.
3. The relationship between *β* and the average information entropy $\bar{H}$ is non-monotonic: When *β* tends towards positive or negative infinity, $\bar{H}$ becomes very small. When −20 < *β* < *−*1, $\bar{H}$ increases with *β*. As *β* increases from −1 to 0, $\bar{H}$ undergoes a weak collapse. A weak upward trend in $\bar{H}$ follows as *β* increases from 0 to 5, and when *β* > 5, $\bar{H}$ collapses until it is close to zero.

By basing our model upon information entropy, it takes into account many parameters that emulate real-life situations, thereby providing a more realistic framework for research into rumor dynamics, the distortion of information, and the fragmentation of opinions. Our results can thus be used to aid network design and decision-making that limits the spread of rumors. For instance, the conservation factor *K* had a pronounced non-linear effect in inhibiting rumor spread, while a high confidence factor *β*, which corresponds to more trust placed in well-connected neighbors, also limited the extent of rumor propagation.
